# Predicting conversion of brain β-amyloid positivity in amyloid-negative individuals

**DOI:** 10.1186/s13195-022-01067-8

**Published:** 2022-09-12

**Authors:** Chae Jung Park, Younghoon Seo, Yeong Sim Choe, Hyemin Jang, Hyejoo Lee, Jun Pyo Kim, for the Alzheimer’s Disease Neuroimaging Initiative

**Affiliations:** 1 Department of Health Sciences and Technology, SAIHST, Sungkyunkwan University, Seoul, South Korea; 2 Alzheimer’s Disease Convergence Research Center, Samsung Medical Center, Seoul, South Korea; 3 Sungkyunkwan University School of Medicine, Seoul, South Korea; 4 Department of Neurology, Samsung Medical Center, Sungkyunkwan University School of Medicine, Seoul, Korea; 5 Neuroscience Center, Samsung Medical Center, Seoul, South Korea; 6 Department of Radiology and Imaging Sciences, Center for Neuroimaging, Indiana University School of Medicine, Indianapolis, 355 W 16th St, Indianapolis, IN 46202 USA

**Keywords:** Alzheimer’s disease, Dementia, Amyloid PET, Prediction model, Machine learning

## Abstract

**Background:**

Cortical deposition of β-amyloid (Aβ) plaque is one of the main hallmarks of Alzheimer’s disease (AD). While Aβ positivity has been the main concern so far, predicting whether Aβ (−) individuals will convert to Aβ (+) has become crucial in clinical and research aspects. In this study, we aimed to develop a classifier that predicts the conversion from Aβ (−) to Aβ (+) using artificial intelligence.

**Methods:**

Data were obtained from the Alzheimer’s Disease Neuroimaging Initiative (ADNI) cohort regarding patients who were initially Aβ (−). We developed an artificial neural network-based classifier with baseline age, gender, *APOE* ε4 genotype, and global and regional standardized uptake value ratios (SUVRs) from positron emission tomography. Ten times repeated 10-fold cross-validation was performed for model measurement, and the feature importance was assessed. To validate the prediction model, we recruited subjects at the Samsung Medical Center (SMC).

**Results:**

A total of 229 participants (53 converters) from the ADNI dataset and a total of 40 subjects (10 converters) from the SMC dataset were included. The average area under the receiver operating characteristic values of three developed models are as follows: Model 1 (age, gender, *APOE* ε4) of 0.674, Model 2 (age, gender, *APOE* ε4, global SUVR) of 0.814, and Model 3 (age, gender, *APOE* ε4, global and regional SUVR) of 0.841. External validation result showed an AUROC of 0.900.

**Conclusion:**

We developed prediction models regarding Aβ positivity conversion. With the growing recognition of the need for earlier intervention in AD, the results of this study are expected to contribute to the screening of early treatment candidates.

**Supplementary Information:**

The online version contains supplementary material available at 10.1186/s13195-022-01067-8.

## Background

The aggregation of β-amyloid (Aβ) peptides into amyloid plaques is one of the main hallmarks of Alzheimer’s disease (AD). The amyloid cascade hypothesis postulates that the accumulation of Aβ plaques initiates the AD pathologic cascade, which triggers the formation of neurofibrillary tangles and neurodegeneration [1, 2]. Consistent with the amyloid cascade hypothesis, recent evidence with in vivo molecular imaging has underlined the association of elevated levels of Aβ with accelerated tau accumulation, cortical atrophy, and cognitive decline in cognitively normal (CN) individuals [3–5]. Furthermore, studies have demonstrated that increased Aβ accumulation could initiate decades prior to the onset of clinical manifestations [6].

Therapeutic trials have developed anti-amyloid treatments for AD individuals with mild dementia or predementia, aiming to reduce Aβ accumulation and prevent cognitive decline. However, despite measurable Aβ reduction, most trials did not show statistical significance in preventing cognitive decline [7–10]. This might be related to the fact that interventions may have been administered too late in the disease progression to exhibit clinical efficacy. Consequently, the concept of primary prevention approaches has emerged. That is, we may need to consider using anti-amyloid therapy in individuals with subthreshold Aβ levels who could convert to Aβ (+) in the future. In fact, a recent study showed that relatively high Aβ levels even in the subthreshold at baseline predicted memory decline and conversion to Aβ (+) status in a subset of Aβ (−) individuals [11]. Accordingly, while Aβ (−) individuals are relatively less focused on both clinical and research-related aspects of AD, the question—“Will this person become Aβ (+) in the near future?”—remains crucial.

Performing primary prevention trials with anti-amyloid agents in subthreshold individuals who are likely to convert to Aβ (+) status could be clinically beneficial. However, it is difficult to identify individuals who are appropriate subjects for the trial. To the best of our knowledge, no classifiers that predict subthreshold individuals who are likely to convert to Aβ (+) status have been developed yet. However, several factors, including age, apolipoprotein E (*APOE*) ε4 allele, and family history, are associated with elevated Aβ levels [12–14]. Individuals with higher Aβ levels, even in the subthreshold at baseline, are more likely to convert to Aβ (+) status in the future. Furthermore, recent findings revealed a focal Aβ elevation in specific brain regions of the Aβ (−) individuals who subsequently converted to Aβ (+) status [15]. Thus, classifiers combining these factors may help identify subjects for the primary prevention trial.

In this study, we aimed to develop a classifier that predicts the patient status conversion from Aβ (−) to Aβ (+) using artificial intelligence. We hypothesized that a combination of age, gender, *APOE* ε4 genotype, family history, and global and regional Aβ uptake could be associated with conversion. We used an artificial neural network (ANN) model that considered different combinations of features to predict the conversion from Aβ (−) to Aβ (+).

## Methods

### Participants

**Fig. 1:**
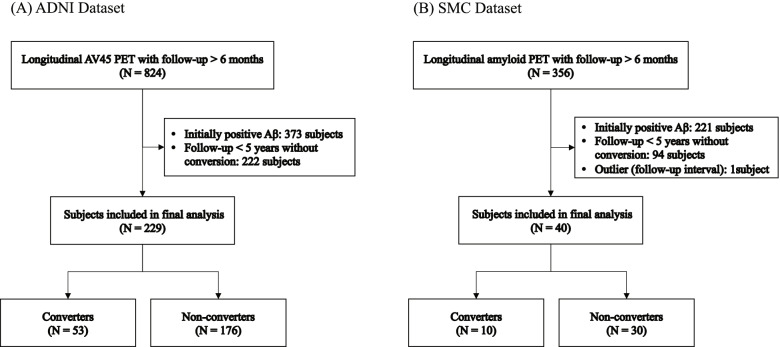
Inclusion and exclusion of the study datasets. **a** Alzheimer’s Disease Neuroimaging Initiative (ADNI) dataset. **b** Samsung Medical Center (SMC) dataset

Data used in this study were obtained from the Alzheimer’s Disease Neuroimaging Initiative (ADNI) cohort. ADNI was launched in 2003 to test whether serial magnetic resonance imaging (MRI), positron emission tomography (PET), other biological markers, and clinical and neuropsychological assessments could be combined to measure the progression of mild cognitive impairment and the early onset of AD. Inclusion and exclusion criteria, clinical and neuroimaging protocols, and other information about ADNI can be found at www.adni-info.org. Demographic information, raw neuroimaging scan data, *APOE* ε4 genotype, and clinical information are publicly available and can be downloaded from the ADNI data repository (www.loni.usc.edu/ADNI/). To develop our prediction models, subjects who underwent longitudinal ^18^F-florbetapir (AV45) PET tests with a total follow-up duration longer than 6 months were selected (*N* = 824). Among these subjects, (1) initially amyloid-positive subjects (*N* = 373) and (2) subjects with a follow-up duration of less than 5 years without conversion to amyloid-positive (*N* = 222) were excluded. We excluded subjects with a short follow-up duration to avoid false-negative. The cutoff for follow-up duration was determined considering the reported mean follow-up time to conversion from Aβ (−) to Aβ (+) [16]. Finally, among the remaining 229 subjects (135 CN, 92 mild cognitive impairment (MCI), 2 dementia), we defined amyloid-negative subjects who converted positive within 5 years as converters (*N* = 53) and subjects who remained amyloid-negative for more than 5 years as non-converters (*N* = 176) (Fig. 1a).

For validation of the prediction model, we recruited subjects who had longitudinal amyloid PET results from the in-house amyloid PET registry of Samsung Medical Center (SMC). A total of 356 subjects had longitudinal Centiloid (CL) data with follow-up duration longer than 6 months, of which 135 were initially Aβ (−). We excluded a subject with a single follow-up visit 8.7 years from the baseline because the long gap between the visits made it difficult to assume the conversion time. After excluding 94 subjects with a follow-up duration of less than 5 years without conversion of Aβ positivity, 40 subjects (6 CN, 28 MCI, 6 dementia) consisting of 10 converters and 30 non-converters were included in the final validation set (Fig. 1b). Because quantification of Aβ burden using the CL method is currently validated and applied only for global uptake data, we validated the prediction model using global SUVR. The institutional review board at SMC approved this study, and informed consent was obtained from the patients and caregivers.

### Image data acquisition and amyloid PET preprocessing

For the analysis of ADNI data, we obtained global and regional ^18^F-florbetapir SUVR values from the UCBERKELEYAV45_11_16_21.csv table downloaded from the ADNI website (http://adni.loni.usc.edu/). ADNI PET acquisition and processing protocols are described elsewhere (www.adni-info.org). Briefly, ^18^F-florbetapir images were co-registered to the MRI image of the subject using SPM8. Following co-registration, images were processed using a FreeSurfer pipeline, which includes skull stripping, segmentation, and delineation of cortical and subcortical regions. Then, the volume-weighted florbetapir mean was extracted from each region and the resulting values were intensity normalized with respect to the whole cerebellum. We used 40 cortical regions (18 frontal, 8 cingulate, 8 lateral parietal, and 6 lateral temporal regions) comprising cortical summary regions according to AV45 processing methods available from the ADNI website for model development. To determine amyloid positivity, we used a whole cerebellum-referenced global SUVR cutoff of 1.11 [17].

SMC participants underwent ^11^C-Pittsburg compound B (PiB), ^18^F-Florbetaben (FBB), or ^18^F-Fluetemetamol (FMM) PET at Samsung Medical Center using a Discovery STe PET/CT scanner (GE Medical Systems, Milwaukee, WI, USA). Following the protocols proposed by the ligand manufacturers, a 30-min emission PET scan 60 min after the injection of a mean dose of 420MBq of PiB or a 20-min emission PET scan 90 min after the injection of a mean dose of 311.5 MBq of FBB or 185 MBq of FMM was performed. To harmonize uptake values across tracers, we calculated CL values based on previous studies regarding SUVR to CL conversion [18–20]. We followed the CL pipelines using SPM8, including co-registration and normalization steps using the cortical target region (CTX-VOI) and the whole cerebellum mask for the reference region. The in-house implementation of the standard CL analysis was validated using the GAAIN PiB data website (http://www.gaain.org). We found excellent correlation between CL values (CL_SMC_ = 1.00 × CL_GAAIN_ − 0.08, R2 = 0.99) [21], showing our pipeline is valid within the acceptance criteria defined by Klunk et al. [18]. After extracting the SUVR values in CTX-VOI, we converted SUVR to CL using each tracer conversion equation (PiB: CL = 100 × (SUVR_PiB_ – 1.009)/1.067, florbetaben: CL = 153.4 × SUVR_FBB_ − 154.9, flutemetamol: CL = 121.42 × SUVR_FMM_ − 121.16). We used a cutoff value of 20 CL, which was reported to be equivalent to FBP SUVR 1.11 [22], to determine the Aβ positivity of SMC subjects.

### Deep-learning models

We developed classifiers utilizing the ADNI dataset that predict the conversion of Aβ positivity within 5 years, which can be predicted by using baseline demographic and neuroimaging information. Three models with various feature combinations were designed: Model 1 was trained with the features of age, gender, and *APOE* ε4 (3 features). Model 2 was trained with features from Model 1 combined with global SUVR (4 features). Model 3 was trained with features from Model 2 combined with 40 regional SUVRs (44 features in total). Categorical features that were converted to were set to 0 or 1, and numerical features were normalized with mean and standard deviation.

Artificial neural network-based models were developed using the PyTorch framework. For the development of the ANN model, we trained the model using the Adam optimizer [23], mean squared error loss function, and ReLU activation function; we also applied batch normalization to prevent internal covariate shift [24]. The grid search approach was used to tune hyperparameters including learning rate, hidden node size, batch size, dropout rate, and weight decay. Stratified 10-fold cross-validation was performed for each model by repeating the random train-validation set splitting 10 times. The models were trained for 100 epochs on graphical processing units (GPUs; NVIDIA GTX 1080Ti).

### Assessment of performance

The performances of the developed classifiers were assessed based on six different metrics: (1) the area under the curve of the receiver operating characteristic (AUROC) curve reflecting the sensitivity and specificity of model predictions; (2) the area under the precision-recall curve (AUPRC), which is a useful performance metric for imbalanced data; (3) sensitivity; (4) specificity; (5) positive predictive value (PPV); and (6) negative predictive value (NPV). The 95% confidence intervals (CIs) of AUROCs and AUPRCs were calculated as well. The averaged values of AUROC and AUPRC were estimated by concatenating all prediction results of each test fold from total 10-fold cross-validation. Sensitivity, specificity, PPV, and NPV values were determined using a classifier threshold of 0.5. After finalizing the model training with the ADNI dataset, external validation with the SMC dataset was performed by loading Model 2. Graphs for the results were plotted using the matplotlib package in Python 3.8.

### Assessment of feature importance

Efforts to interpret machine learning models have been continued, and model-agnostic methods have been suggested [25–27]. The feature importance in Model 3 was analyzed regarding model interpretability using the Captum Python library, which can describe internals in the PyTorch-based model [27]. Specifically, we applied the integrated gradient method to estimate the attributes of the prediction of an ANN with respect to certain inputs [28]. We averaged the attribution scores across the test sets to derive a representative value of the feature importance for comparison. For visualization of feature importance, we used the ggseg R package, which can visualize the Desikan-Killiany ROI-wise values.

### Statistical analysis

We compared the characteristics of converters and non-converters using Student’s *t*-test for continuous variables and the chi-square test for categorical variables. All tests were two-sided and considered statistically significant at *p* < 0.05. Statistical analyses were performed using the scipy package of Python 3.8.

## Results

### Clinical characteristics

Table 1 describes the characteristics of the clinical and PET results of converters and non-converters. A total of 229 participants from the ADNI dataset were included in model development. A total of 53 participants (23.1%) were converted to beta-amyloid-positive within 5 years, while 176 remained negative. Statistical tests underlined significant group differences in *APOE* ɛ4 carrier status and global SUVR (*p* < 0.05). With respect to SMC participants, a total of 10 out of 40 subjects (25.0%) were converted to Aβ (+) within 5 years, while 30 subjects remained negative. Statistical tests highlighted significant group differences in age and global CL (*p* < 0.05).

**Table 1:** Clinical characteristics of β-amyloid positivity converters and non-converters

	ADNI dataset				SMC dataset				***p***-value^†^
	Total	Converter	Nonconverter	***p***-value	Total	Converter	Nonconverter	***p***-value	
Subjects, *N* (%)	229 (100.0)	53 (23.1)	176 (75.1)	-	40 (100.0)	10 (25.0)	30 (75.0)	-	-
Follow-up duration (years)	7.1 (1.7)	5.7 (2.2)	7.5 (1.3)	<0.001*	6.1 (2.0)	3.9 (1.2)	6.7 (1.6)	<0.001*	<0.001*
Age, mean years (SD)	71.7 (7.3)	72.9 (6.8)	71.3 (7.5)	0.163	70.1 (7.7)	75.0 (8.1)	69.5 (7.3)	0.050	0.498
Female, *N* (%)	108 (47.2)	23 (43.4)	85 (48.3)	0.639	28 (68.3)	7 (70.0)	20 (67.7)	1.000	0.028*
*APOE* ε4 carriers, *N* (%)	54 (23.6)	20 (37.7)	34 (19.3)	0.010*	5 (12.5)	3 (30.0)	2 (6.7)	0.168	0.175
Education, years (SD)	16.5 (2.7)	16.6 (2.3)	16.5 (2.8)	0.813	9.0 (4.6)	11.2 (4.2)	8.3 (4.6)	0.087	<0.001*
Family history, *N* (%)	124 (54.1)	31 (58.4)	93 (52.8)	0.571	10 (24.4)	4 (40.0)	6 (19.4)	0.369	0.001*
Amyloid tracer uptake^a^, mean (SD)	1.017 (0.054)	1.058 (0.039)	1.005 (0.052)	<0.001*	3.377 (7.325)	12.052 (5.492)	0.088 (4.754)	<0.001*	-

*ADNI* Alzheimer’s Disease Neuroimaging Initiative, *SMC* Samsung Medical Center, *SD* standard deviation, *APOE ε4* apolipoprotein E ε4, *SUVR* standardized uptake value ratios, *AD* Alzheimer’s disease

^†^Comparisons between cohorts

^a^Values represent global SUVR in the ADNI dataset and global Centiloid in the SMC dataset

*Statistically significant (*p* < 0.05)

### Model performance

Table 2 reports the performances of each model. The AUROC and AUPRC after 10 times repeated 10-fold cross-validation were described in the mean and 95% CI. Model 3 with features of age, gender, *APOE* ɛ4 carrier, global SUVR, and regional SUVR demonstrated the highest mean performance of all models after repeated cross-validation: The mean AUROC was 0.841 (95% CI 0.832–0.849) and the mean AUPRC was 0.627 (95% CI 0.610–0.645). Sensitivity of 0.600 (95% CI 0.581–0.619), specificity of 0.869 (95% CI 0.862–0.875), PPV of 0.579 (95% CI 0.562–0.597), and NPV of 0.878 (95% CI 0.873–0.884) were obtained. The fitted hyperparameters at the highest performance were as follows: a first hidden layer of 256 nodes, a second hidden layer of 32 nodes, a batch size of 8, a learning rate of 0.0003, a dropout rate of 0.3, and a weight decay of 0.0001. The inclusion of family history as a predictor did not increase the model performances (results not shown).

**Table 2:** Beta-amyloid positivity classifier performances. Three different models were developed with different feature combinations

Dataset	Model	Features^a^	AUROC (95% CI)	AUPRC (95% CI)	Sensitivity (95% CI)	Specificity (95% CI)	PPV (95% CI)	NPV (95% CI)
A. ADNI (development set)	Model 1	Age, gender, *APOE* ε4 carriers (3)	0.674 (0.666–0.683)	0.374 (0.364–0.384)	0.606 (0.595–0.616)	0.692 (0.668–0.715)	0.373 (0.355–0.392)	0.853 (0.849–0.858)
	Model 2	Age, gender, *APOE* ε4 carriers, global SUVR (4)	0.814 (0.806–0.821)	0.549 (0.534–0.564)	0.744 (0.707–0.780)	0.727 (0.690–0.764)	0.454 (0.430–0.478)	0.905 (0.896–0.913)
	Model 3	Age, gender, *APOE* ε4 carriers, global SUVR, regional SUVR (44)	0.841 (0.832–0.849)	0.627 (0.610–0.645)	0.600 (0.581–0.619)	0.869 (0.862–0.875)	0.579 (0.562–0.597)	0.878 (0.873–0.884)
B. SMC (external validation set)	Model 2	Age, gender, *APOE* ε4 carriers, global SUVR (4)	0.900	0.625	1.000	0.700	0.526	1.000

Measurements were described as averages and the 95% confidence interval of 10 times repeated 10-fold cross-validation with the Alzheimer’s Disease Neuroimaging Initiative (ADNI) dataset. The Samsung Medical Center (SMC) dataset was used for external validation to test the Model 2.

*AUROC* area under the receiver operating characteristic, *AUPRC* area under the precision-recall curve, *CI* confidence interval, *PPV* positive predictive value, *NPV* negative predictive value, *ADNI* Alzheimer’s Disease Neuroimaging Initiative, *SMC* Samsung Medical Center, *APOE ε4* apolipoprotein E ε4, *SUVR* standardized uptake value ratio

^a^Numbers in parentheses indicate the total number of features used in each model

**Fig. 2:**
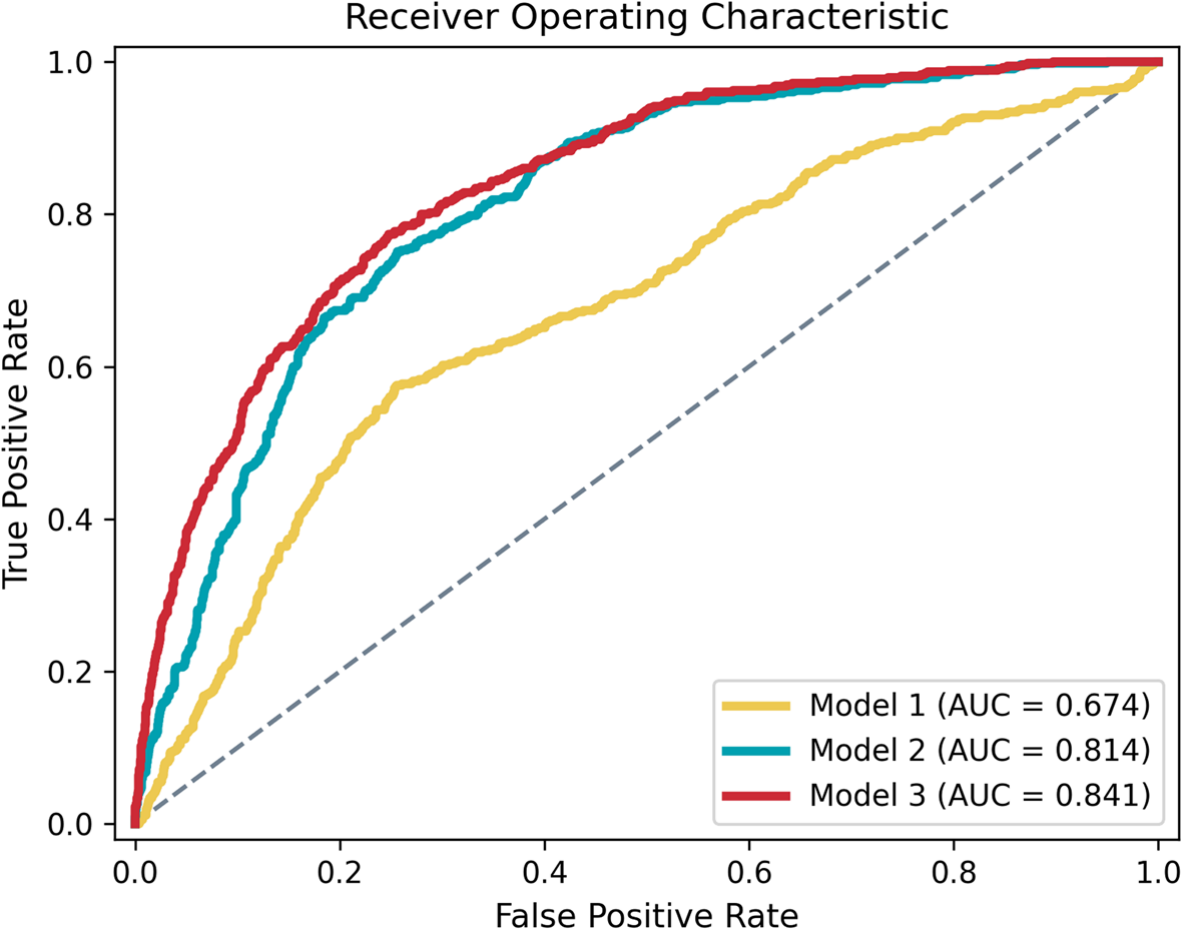
Receiver operating characteristic curves of three artificial neural network models that classify the β-amyloid positivity within 5 years. Mean curves of 10 times repeated 10-fold cross-validation are plotted. Each model included the following features for training with the Alzheimer’s Disease Neuroimaging Initiative (ADNI) dataset: Model 1: age + gender + *APOE* ε4 carriers, Model 2: features from Model 1 + global SUVR, and Model 3: features from Model 2 + regional SUVR

Model 2, which was trained with demographic and SUVR data, had a mean AUROC of 0.814 (95% CI 0.806–0.821) and a mean AUPRC of 0.549 (95% CI 0.534–0.564). Model 1, trained without PET data, which included only age, gender, and *APOE* ɛ4, showed a mean AUROC of 0.674 (95% CI 0.666–0.683) and mean AUPRC of 0.374 (95% CI 0.364–0.384). AUROC curves of the three models are plotted in Fig. 2. DeLong test results showed that Model 3 had higher AUROC than Model 2 (*p* = 0.003) or Model 1 (*p* < 0.001). The external validation results using the SMC dataset were as follows: AUROC of 0.900, AUPRC of 0.625, sensitivity of 1.000, specificity of 0.700, PPV of 0.526, and NPV of 1.000. All performance metrics are listed in Table 2.

### Model interpretability

**Fig. 3:**
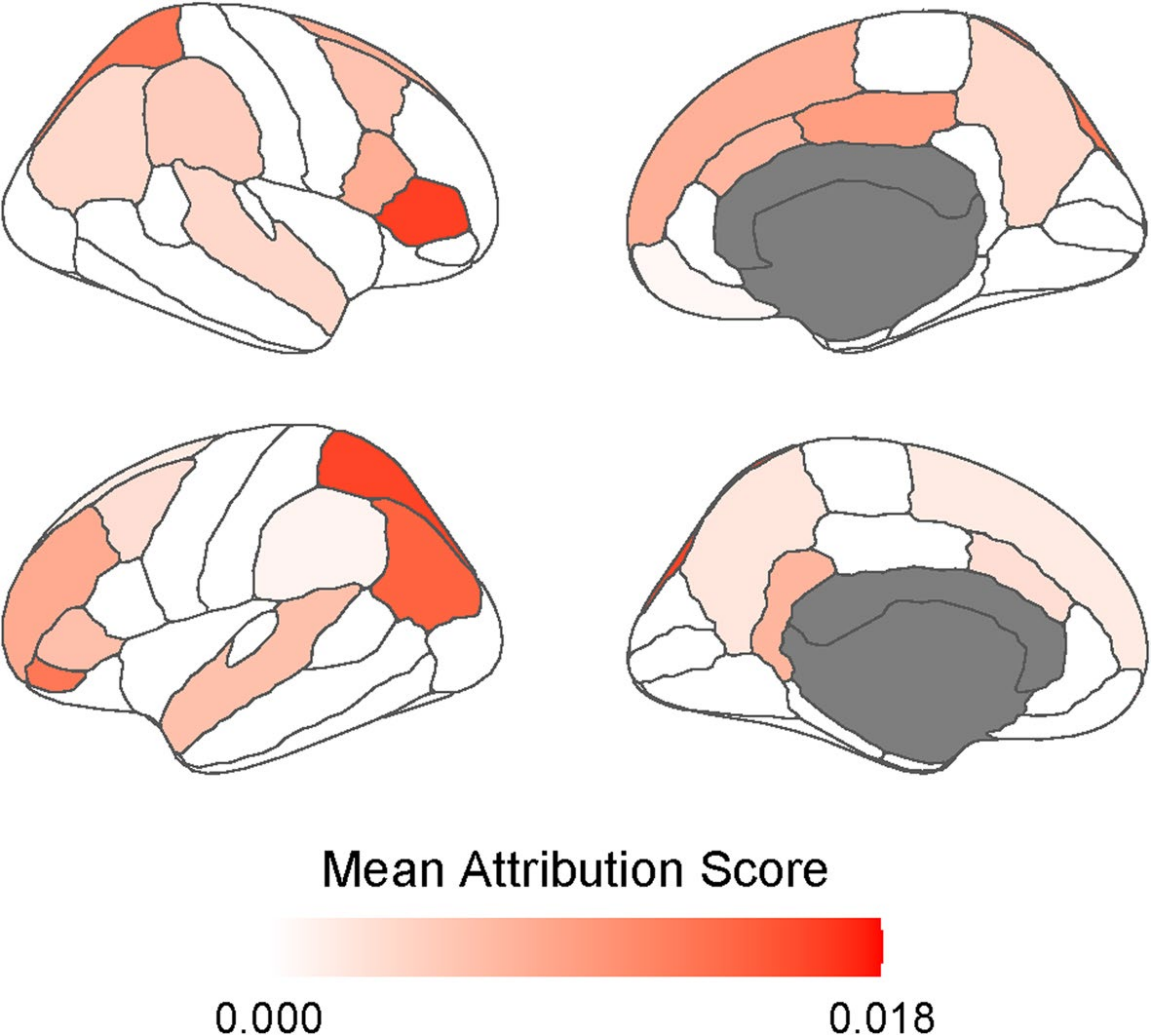
Visualization of feature importance using mean attribution scores

The feature attribution to the classifier output probability was estimated by loading the best-performing model for each fold in the experimental setup for model 3. Feature-wise averaging of 10-fold results and sorting features positively contributing to the prediction of converters were performed. Accordingly, the top 12 features among those average values are presented in Table 3. The results show features contributing to the prediction of subjects as Aβ converters. The features highly contributing included global SUVR, *APOE* ɛ4 carrier status, regional SUVRs of the right pars triangularis, left lateral parietal cortex, and left frontal pole. The mean attribution scores are shown in Fig. 3. Overall, the medial and lateral parietal and frontal cortices, as well as superior temporal and cingulate cortices, exhibited high attribution scores.

**Table 3:** Features attributing to the classification of β-amyloid positivity conversion in Model 3

Feature	Importance
Global SUVR	0.025
*APOE* e4 carrier	0.019
Right pars triangularis	0.016
Left superior parietal	0.016
Left inferior parietal	0.014
Left frontal pole	0.012
Right superior parietal	0.012
Left pars orbitalis	0.012
Right posterior cingulate	0.009
Left rostral middle frontal	0.008
Right pars opercularis	0.008
Right superior frontal	0.007

*Aβ* beta-amyloid, *APOE ε4* apolipoprotein E ε4, *SUVR* standardized uptake value ratio

## Discussion

In this study, we developed classifiers that predicted the conversion of patient status from Aβ (−) to Aβ (+) using baseline information on demographics and neuroimaging test results from the ADNI database. The major findings of this study are as follows. First, the incidence of conversion to amyloid positivity was 23.1% (53/229) within 5 years. Second, age, gender, and *APOE* genotype, but not family history, were effective in predicting the conversion to amyloid positivity. Finally, the prediction model, which consisted of age, gender, *APOE* genotype, and global SUVR, showed good accuracy (AUROC = 0.814). Furthermore, the addition of regional SUVR led to an improvement in the prediction performance (AUROC = 0.841). Our findings highlight the distinctive features that should be taken into consideration when selecting candidates for primary prevention treatment in CN individuals.

Our first major finding was that the incidence rate of conversion to amyloid positivity was 23.1% within 5 years, as 53 out of 229 participants were Aβ converters. Our findings are consistent with those of the previous studies. Specifically, recent studies from different cohorts have reported the annual incidence of conversion to Aβ (+) among elderly Aβ (−) CN individuals, ranging from 3.1 to 13% [29, 30]. Thus, approximately 20% of baseline Aβ (−) individuals converted to Aβ (+) status, suggesting that these individuals need to be considered candidates for primary prevention.

Age, gender, *APOE* genotype, and family history are well-known risk factors for amyloid positivity. However, in the present study, age, gender, and *APOE* genotype, but not family history, were predictive of conversion to amyloid positivity. Our findings are in line with previous studies reporting factors associated with the rate of Aβ accumulation, such as *APOE* genotype [29], age, and sex [16] in Aβ (−) individuals. However, our findings contradict a previous study, which reported a lack of obvious differentiating demographic features between amyloid converters and non-converters [30]. The discrepancy could be attributed to the differences in study designs between the reference and the present study, given the limited follow-up (median imaging follow-up was 1.3 years) and considerably smaller sample size of baseline Aβ (−) subjects (123 vs. 229).

Our third major finding was that the prediction model, which consists of age, gender, *APOE* genotype, and global SUVR, showed good performance (AUROC = 0.814). Note that despite being in the subthreshold range, the inclusion of global SUVR increased the performance. A few recent studies on longitudinal amyloid PET imaging have shown that the annual change rate of Aβ is biphasic [6, 31, 32]. The deflection point of this biphasic curve is known to be higher than the Aβ threshold. In subjects with baseline SUVR lower than this deflection point, the rate of Aβ accumulation increases as the baseline SUVR increases. In line with this pattern, our findings showed that the relative proximity of global SUVR to the Aβ threshold is a crucial factor in the classification of Aβ (−) subjects into converters and non-converters. External validation with the model using demographic features and global SUVR showed excellent performance (AUROC = 0.900), which means that the developed model can be useful in the clinic by discerning candidates who might convert to Aβ (+).

Moreover, the addition of regional SUVR led to an improvement in the prediction performance (AUROC = 0.841). This result is consistent with findings from previous studies that suggest that individuals with focal Aβ accumulation and negative global SUVR demonstrated early clinical and neuroimaging features of AD progression [33, 34]. Of note, the increased specificity and decreased sensitivity indicate that the model becomes more stringent with the incorporation of regional SUVR. It can be inferred that the model filters out subjects with high baseline SUVR but less risky regional uptake patterns. However, this needs further validation in a dataset with regional uptake values available. The incorporation of regional SUVR values could provide a more detailed understanding of the relationship between regionally specific amyloid aggregation and amyloid-related neurodegenerative changes. In the present study, the combined model that used demographic features, global SUVR, and regional SUVR resulted in the best performance, suggesting a better capacity to predict the conversion of Aβ positivity compared to other models tested. To our knowledge, no studies have developed machine learning classifiers to predict amyloid conversion in Aβ (−) subjects.

This study identifies the list of highly influencing features: global SUVR and *APOE* ɛ4 carrier status contributed the most to the prediction of Aβ conversion, followed by regional SUVRs in the medial and lateral parietal, medial and lateral frontal, and cingulate cortices. Our results are in line with previous studies on the early involvement pattern of cortical Aβ accumulation [35]. Thus, our model is likely to capture early AD patterns of amyloid PET.

Overall, the model developed in this study was able to predict the conversion of Aβ positivity in Aβ (−) subjects. The model performance improved with the inclusion of global and regional SUVRs and achieved good performance, which was validated in an independent dataset.

### Limitations

A few limitations of our study need to be noted. First, the sample sizes of the datasets used in our study were modest. In fact, the ADNI is the cohort with the largest number of longitudinal amyloid PET data. However, the SMC dataset had a smaller number of eligible subjects especially when limited to initially negative subjects who had longitudinal PET data. Despite the limitations of the sample size, we were able to validate our results. Accumulation of amyloid PET data, especially in amyloid-negative individuals, is needed for a more robust validation of our results. Second, while model 3 showed the best performance, the sensitivity and positive predictive value were relatively low. We can adjust the prediction score threshold of the neural network model to find a different balance between specificity and sensitivity depending on the purpose of the prediction model. In contrast to the results in the ADNI cohort, the specificity was relatively low in the SMC cohort. The difference may be attributed to the smaller number of subjects or differences between the cohorts, such as ethnicity, gender, educational attainment, and family history. Since the false positivity may pose ethical challenges in applying the model in clinical trials, the prediction score threshold of the model may have to be adjusted in favor of specificity rather than sensitivity. More importantly, incorporating additional features such as neuropsychological test results, other neuroimaging phenotypes, or genetic factors is needed to improve the overall performance of the model. Third, we could not test the model including regional SUVR with the SMC dataset, although the model showed the best performance in the ADNI cohort. SMC subjects were recruited from a PET registry comprising amyloid PET scans of three different tracers, which forced us to use the CL method for harmonization. Unfortunately, the application of the CL method for regional uptake has not yet been validated. Once the methodology regarding the regional application of the CL is validated, it needs to be tested. Fourth, we used CL values to validate a model developed using global SUVR values. While this was possible since CL values have a strong linear relationship with FBP SUVR [36], further validation studies with harmonized features are warranted. Nevertheless, it is noteworthy that we developed well-performing models for the prediction of Aβ conversion and found important features that should be considered for the selection of primary prevention of AD.

## Conclusion

We developed prediction models for the prediction of Aβ positivity conversion, which showed good prediction performance and coherence with the previously known nature of Aβ pathology. With the growing recognition of the need for earlier intervention in AD, the results of this study are expected to contribute to the screening of early treatment candidates.

## Supplementary Information

**Additional file 1: Supplementary Table 1.** Model performances according to different cutoff for follow-up duration. **Supplementary Table 2.** Model performances according to different Aβ cutoff values. **Supplementary Figure 1.** Longitudinal β-amyloid positivity trajectories. Amyloid tracer uptake values versus follow-up years are plotted for (A) Alzheimer’s Disease Neuroimaging Initiative (ADNI) dataset and (B) Samsung Medical Center (SMC) dataset. Trajectory of converters are colored in scarlet, whereas non-converters are colored in gray. The dotted horizonal line is the cutoff for β-amyloid positivity, where SUVR 1.11 for ADNI and Centiloid of 20 for SMC dataset, respectively.

## Data Availability

Data used in the preparation of this article were obtained from the Alzheimer’s Disease Neuroimaging Initiative (ADNI) database (http://adni.loni.usc.edu). As such, the investigators within the ADNI contributed to the design and implementation of ADNI and/or provided data but did not participate in the analysis or writing of this report. A complete listing of ADNI investigators can be found at: http://adni.loni.usc.edu/wp-content/uploads/how_to_apply/ADNI_Acknowledgement_List.pdf.

## Abbreviations

Aβ: Beta-amyloid
AD: Alzheimer’s disease
ADNI: Alzheimer’s Disease Neuroimaging Initiative
ANN: Artificial neural network
*APOE* ε4: Apolipoprotein E ε4
AUPRC: Area under the precision-recall curve
AUROC: Area under the receiver operating characteristic
CI: Confidence interval
CL: Centiloid
CN: Cognitively normal
FBB: ^18^F-Florbetaben
FMM: ^18^F-Fluetemetamol
MCI: Mild cognitive impairment
MRI: Magnetic resonance imaging
NPV: Negative predictive value
PET: Positron emission tomography
PiB: ^11^C-Pittsburg compound B
PPV: Positive predictive value
SMC: Samsung Medical Center
SUVRs: Standardized uptake value ratios
