# Supplementary material for: Predicting conversion of brain β-amyloid positivity in amyloid-negative individuals
Source: Alzheimers Res Ther. 2022 Sep 12;14:129. doi: 10.1186/s13195-022-01067-8 (PMC9465850; doi:10.1186/s13195-022-01067-8)

**Supplementary Table 1.** Model performances according to different cutoff for follow-up duration

|  | AUROC (95%CI) | |
| --- | --- | --- |
|  | 3 years | 5 years |
| Model 1 | 0.599 (0.577-0.621) | 0.676 (0.664-0.688) |
| Model 2 | 0.848 (0.840-0.855) | 0.814 (0.806-0.821) |
| Model 3 | 0.882 (0.871-0.893) | 0.841 (0.832-0.849) |

*AUROC* area under the receiver operating characteristic curve, *CI* confidence interval

**Supplementary Table 2.** Model performances according to different Aβ cutoff values

| Cutoff | AUROC (95% CI) |
| --- | --- |
| SUVR 1.07 (CL 12) | 0.827 (0.818-0.835) |
| SUVR 1.11 (CL 20) | 0.839 (0.832-0.849) |
| SUVR 1.16 (CL 30) | 0.893 (0.884-0.902) |

*AUROC* area under the receiver operating characteristic curve, *CI* confidence interval, *CL* Centiloid

**Supplementary Figure 1.** Longitudinal β-amyloid positivity trajectories. Amyloid tracer uptake values versus follow-up years are plotted for (A) Alzheimer’s Disease Neuroimaging Initiative (ADNI) dataset and (B) Samsung Medical Center (SMC) dataset. Trajectory of converters are colored in scarlet, whereas non-converters are colored in gray. The dotted horizonal line is the cutoff for β-amyloid positivity, where SUVR 1.11 for ADNI and Centiloid of 20 for SMC dataset, respectively


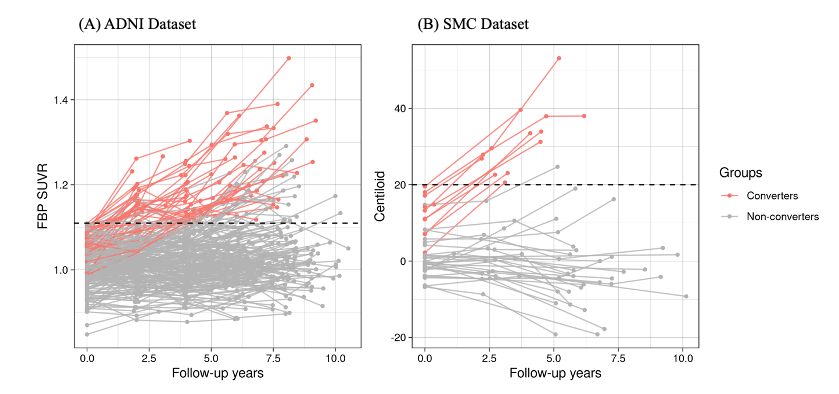

Supplement: Supplementary file 1 — Additional file 1: Supplementary Table 1. Model performances according to different cutoff for follow-up duration. Supplementary Table 2. Model performances according to different Aβ cutoff values. Supplementary Figure 1. Longitudinal β-amyloid positivity trajectories. Amyloid tracer uptake values versus follow-up years are plotted for (A) Alzheimer’s Disease Neuroimaging Initiative (ADNI) dataset and (B) Samsung Medical Center (SMC) dataset. Trajectory of converters are colored in scarlet, whereas non-converters are colored in gray. The dotted horizonal line is the cutoff for β-amyloid positivity, where SUVR 1.11 for ADNI and Centiloid of 20 for SMC dataset, respectively. [file 13195_2022_1067_MOESM1_ESM.docx]
